# Effects of Intra-Amniotic Lipopolysaccharide and Maternal Betamethasone on Brain Inflammation in Fetal Sheep

**DOI:** 10.1371/journal.pone.0081644

**Published:** 2013-12-17

**Authors:** Elke Kuypers, Reint K. Jellema, Daan R. M. G. Ophelders, Jeroen Dudink, Maria Nikiforou, Tim G. A. M. Wolfs, Ilias Nitsos, J. Jane Pillow, Graeme R. Polglase, Matthew W. Kemp, Masatoshi Saito, John P. Newnham, Alan H. Jobe, Suhas G. Kallapur, Boris W. Kramer

**Affiliations:** 1 Department of Pediatrics, School of Mental Health and Neuroscience, Maastricht University Medical Center, Maastricht, The Netherlands; 2 Department of Pediatrics, Erasmus Medical Center-Sophia, Rotterdam, The Netherlands; 3 School of Women’s and Infants’ Health, The University of Western Australia, Perth, Australia; 4 The Ritchie Centre, Monash Institute of Medical Research, Melbourne, Australia; 5 School of Anatomy, Physiology and Human Biology, The University of Western Australia, Perth, Australia; 6 Division of Pulmonary Biology, Cincinnati Children's Hospital Medical Center, Cincinnati, Ohio, United States of America; Friedrich-Alexander-University Erlangen, Germany

## Abstract

**Rationale:**

Chorioamnionitis and antenatal glucocorticoids are common exposures for preterm infants and can affect the fetal brain, contributing to cognitive and motor deficits in preterm infants. The effects of antenatal glucocorticoids on the brain in the setting of chorioamnionitis are unknown. We hypothesized that antenatal glucocorticoids would modulate inflammation in the brain and prevent hippocampal and white matter injury after intra-amniotic lipopolysaccharide (LPS) exposure.

**Methods:**

Time-mated ewes received saline (control), an intra-amniotic injection of 10 mg LPS at 106d GA or 113d GA, maternal intra-muscular betamethasone (0.5 mg/kg maternal weight) alone at 113d GA, betamethasone at 106d GA before LPS or betamethasone at 113d GA after LPS. Animals were delivered at 120d GA (term=150d). Brain structure volumes were measured on T2-weighted MRI images. The subcortical white matter (SCWM), periventricular white matter (PVWM) and hippocampus were analyzed for microglia, astrocytes, apoptosis, proliferation, myelin and pre-synaptic vesicles.

**Results:**

LPS and/or betamethasone exposure at different time-points during gestation did not alter brain structure volumes on MRI. Betamethasone alone did not alter any of the measurements. Intra-amniotic LPS at 106d or 113d GA induced inflammation as indicated by increased microglial and astrocyte recruitment which was paralleled by increased apoptosis and hypomyelination in the SCWM and decreased synaptophysin density in the hippocampus. Betamethasone before the LPS exposure at 113d GA prevented microglial activation and the decrease in synaptophysin. Betamethasone after LPS exposure increased microglial infiltration and apoptosis.

**Conclusion:**

Intra-uterine LPS exposure for 7d or 14d before delivery induced inflammation and injury in the fetal white matter and hippocampus. Antenatal glucocorticoids aggravated the inflammatory changes in the brain caused by pre-existing intra-amniotic inflammation. Antenatal glucocorticoids prior to LPS reduced the effects of intra-uterine inflammation on the brain. The timing of glucocorticoid administration in the setting of chorioamnionitis can alter outcomes for the fetal brain.

## Introduction

Preterm birth (before 37 weeks of gestation) is associated with chorioamnionitis, an infection/inflammation of the amniotic fluid and placental membranes, which is present in up to 60% of early gestation preterm births [1,2]. Exposure to intra-uterine inflammation is associated with adverse effects on fetal lung [3,4], gut [5,6] and brain [7] development and increases the risk for complications in postnatal life [8]. Administration of antenatal glucocorticoids to induce fetal lung maturation has become standard of care for mothers at risk of preterm delivery, irrespective of the cause of preterm birth [9,10]. As chorioamnionitis is often clinically silent prior to preterm labor, combined exposures to antenatal glucocorticoids and chorioamnionitis are very common in preterm fetuses. 

There is a clear relationship between the presence of chorioamnionitis and the development of neurological complications in preterm infants [11,12]. Exposure to intra-uterine inflammation can lead to intraventricular hemorrhage and white matter injury (WMI) which can manifest clinically as periventricular leukomalacia (PVL), cerebral palsy (CP) [13,14] and an increased risk for other adverse neurodevelopmental complications such as cognitive impairments [15], autism spectrum disorders [16,17] and schizophrenia [18,19]. Animal models of intra-uterine inflammation have helped to reveal some of the molecular mechanisms underlying fetal brain injury [20-22]. Chronic intra-amniotic administration of lipopolysaccharide (LPS) to fetal sheep induced microglial activation and white matter injury in the subcortical white matter (SCWM) [23]. Gavilanes et al. showed that a single intra-amniotic bolus of LPS resulted in microglial activation, astrocyte proliferation and increased apoptosis in the ovine fetal brain, which were associated with functional changes in EEG after preterm birth [24]. 

The effects of intra-uterine inflammation are not limited to the brain [8]. We showed that intra-amniotic LPS induced lung inflammation and activation of the fetal immune system in sheep [25-27]. Antenatal glucocorticoid administration before or after the LPS exposure attenuated inflammation and injury in the lung and reduced the fetal immune response [25,28]. In the current study, we hypothesized that antenatal glucocorticoids would modulate inflammation in the fetal near term brain and prevent subsequent hippocampal and white matter injury after intra-amniotic LPS exposure. For this purpose, fetal sheep were exposed to intra-amniotic LPS at 2 different gestational ages (GA) before preterm delivery to investigate the acute and persistent effects of the exposures on the near term brain. The effect of antenatal glucocorticoid treatment was evaluated by administration of maternal betamethasone before the LPS exposure (i.e. betamethasone pre-treatment) to model the clinical situation for women who present with preterm labor and receive glucocorticoid treatment prior to the infectious exposure, often following rupture of membrane. We also gave betamethasone after the LPS exposure (i.e. betamethasone post-treatment) at 7-day intervals as a representative of the clinical scenario of women in preterm labor due to chronic indolent chorioamnionitis which then receive antenatal glucocorticoids. An interval of 7 days between the two exposures was chosen as representative of the period between recognition of preterm labor and the actual preterm delivery [1] and the repeated administration of antenatal glucocorticoids at weekly intervals [29].

## Materials and Methods

### Animal study

All studies were approved by the Animal Ethics Committees at The University of Western Australia and Cincinnati Children`s Hospital Medical Center (animal ethics protocol RA/3/100/830). The experimental design of this animal study was published previously [26]. Briefly, time-mated ewes with singleton fetuses were exposed to LPS (intra-amniotic injection (IA) of 10 mg LPS *Escherichia Coli*, 055:B5 Sigma-Aldrich, St. Louis, MO, USA) to induce intra-amniotic inflammation and/or maternal betamethasone (intra-muscular injection (IM) of betamethasone as Celestone Soluspan, Schering-Plough, North Ryde, New South Wales, Australia at a dose of 0.5 mg/kg maternal weight which has been demonstrated to induce lung responses in sheep [30,31]) either at 106d GA or 113d GA. The following are the treatment groups: IA LPS at 113d GA (7d LPS group; n=7), IM betamethasone at 113d GA (7d Beta group; n=6), IM betamethasone at 106d GA followed by IA LPS at 113d GA (Beta-Pre + 7d LPS group; n=6), IA LPS at 106d GA (14d LPS group; n=7) or IA LPS at 106d GA followed by IM betamethasone at 113d GA (14d LPS + Beta-Post group; n=6). Control animals were exposed to IA saline and IM saline at 106d and 113d GA (Table 1). 

**Table 1:** Experimental treatment groups.

	Injection at **106d** GA	Injection at **113d** GA	Number of animals
Control	IA saline	IM saline	4
7d LPS	-	IA LPS	7
7d Beta	-	IM Beta	6
Beta-Pre + 7d LPS	IM Beta	IA LPS	6
14d LPS	IA LPS	-	7
14d LPS + Beta-Post	IA LPS	IM Beta	6

IA: intra-amniotic; IM: intra-muscular; GA: gestational age; Beta: betamethasone; LPS: lipopolysaccharide; Beta-Pre: betamethasone pre-treatment; Beta-Post: betamethasone post-treatment.

As betamethasone can induce preterm delivery in sheep, all ewes also received a single intra-muscular injection of medroxyprogesterone acetate (Depo-Provera 150 mg, Kenral, New South Wales, Australia) at 100 days GA to decrease the risk of preterm labor. Lambs were delivered by caesarean section at 120d GA (term = 150d GA) and euthanized at birth by an intra-venous injection of 100 mg/kg pentobarbital. The gestational age of 120 days is comparable to a near term infant in terms of brain development [22,32,33]. Animal characteristics and cord blood values at birth were reported previously and did not differ between groups [26]. The fetal brain was perfusion-fixed via the carotid arteries in 0.1M phosphate buffer (pH 7.4) and with 4% paraformaldehyde at 4°C for 24 hours and then stored in 30% sucrose at 4°C. 

### MRI analysis

After perfusion fixation, anatomical T2-weighted images of the fetal brains in the skull were acquired using a 7 Tesla Bruker Biospin 70/30 USR scanner (Bruker, Ettlingen, Germany). After optimization all T2-weighted images were acquired using spin-echo sequences with the following parameters: (TR=2000 and TE=78 ms), with an isovolumetric voxel size of 200 µm^3^ and no gap. The field of view (FOV) was 55x70x45 mm and scan matrix size 275x512x225 mm.

**Figure 1:**
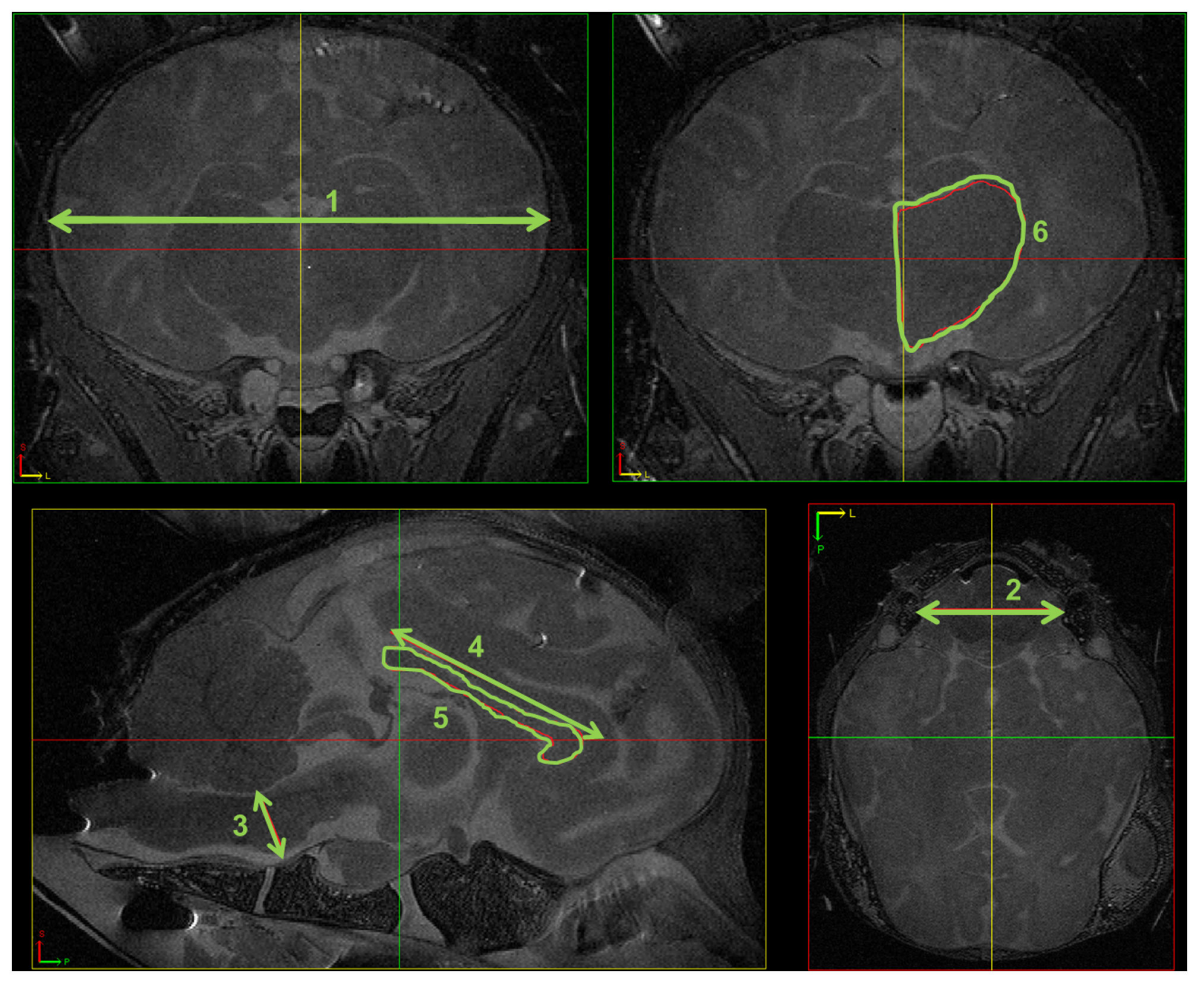
Brain volumes measured with MRI images. The mid-sagittal antero-posterior brain diameter (mm) (1), mid-sagittal cranio-caudal diameter of the cerebellum (mm) (2), mid-sagittal antero-posterior diameter of the pons (mm) (3), mid-sagittal antero-posterior length of the corpus callosum (mm) (4), mid-sagittal volume of the corpus callosum (mm^3^) (5) and maximal parasagittal volume of thalamus (mm^3^) (6) of the ovine fetal brain were measured on T2-weighted MRI images.

MRI data were processed using the MIPAV software package (Medical Image Processing, Analysis, and Visualization, version 5.2.1; National Institutes of Health), which enables quantitative analysis and visualization of medical images. A blinded observer measured size and volume of several brain structures in T2-weighted images on fixation artefact free slices. Measurements included: (1) mid-sagittal antero-posterior brain diameter (mm), (2) mid-sagittal cranio-caudal diameter of the cerebellum (mm), (3) mid-sagittal antero-posterior diameter of the pons (mm) (4), mid-sagittal antero-posterior length of the corpus callosum (mm) (5), mid-sagittal volume of the corpus callosum (mm^3^) and (6) maximal parasagittal volume of thalamus (mm^3^) (Figure 1).

### Immunohistochemistry

For immunohistochemistry of the fetal brain, tissue from the right hemisphere was embedded in 10% gelatin. Serial coronal sections, with a thickness of 50 µm to maintain the cellular morphology [34], were cut on a vibrotome (Leica Biosystems, Nussloch, Germany). Sections were stained by free-floating techniques as described previously [34] for ionized calcium binding adaptor molecule 1 (IBA1, #019-19741, Wako Pure Chemical Industries, Osaka, Japan), glial fibrillary acidic protein (GFAP, Z0334, Dakocytomation, Glostrup, Denmark) cleaved caspase-3 (Asp175, #9661S, Cell Signaling Technology, Boston, USA), Ki67 (M7240, Dakocytomation), myelin basic protein (MBP, MAB386, Merck Millipore, Billerica, MA, USA) and synaptophysin (MAB5258, Merck Millipore). Briefly, sections were rinsed with Tris buffered saline (TBS, pH 7.6) and TBS-Triton (TBS-T, pH 7.6). Endogenous peroxidase activity was blocked by incubating in 0.3% H_2_O_2_ in TBS. Next, the sections were incubated overnight at 4°C with the diluted primary antibody (IBA1 1:1000, GFAP 1:2000, cleaved caspase-3 1:800, Ki67 1:100, MBP 1:2000, synaptophysin 1:2000 with 0.3% donkey serum). After rinsing, a secondary donkey-anti-rabbit (IBA1, GFAP, cleaved caspase-3), donkey-anti-mouse (synaptophysin, Ki67) or donkey-anti-rat (MBP) biotin labeled antibody was added for incubation in room temperature for 2 hours. The immunostaining was enhanced with Vectastain ABC peroxidase Elite kit (PK-6200, Vector Laboratories, Burlingame, USA) followed by nickel sulfate-diaminobenzidine. The stained sections were mounted on gelatin-coated glass slides, dehydrated and coverslipped.

### Quantification

For the analysis of IBA1 and GFAP immuno-reactivity in the hippocampus, one digital image per coronal section (n=5 sections per animal) of the dentate gyrus (DG), cornu ammonis (CA)1/2, CA3 and CA4 were acquired at 100x magnification with an Olympus AX-70 microscope connected to a digital camera (F-view, Olympus, Tokyo, Japan). In the SCWM and PVWM two images per coronal section (n=5 sections per animal) were obtained on a standardized location within these regions of interest. All images were collected under the same lighting conditions. The area fraction (%) of IBA1 or GFAP immuno-reactivity was measured by applying a standard threshold using a specifically designed algorithm in Leica QWin Pro V 3.5.1 software (Leica, Rijswijk, the Netherlands) [34].

To measure the density (cells per mm^2^) of Ki67-positive cells and cleaved caspase-3 positive cells, digital images of the hippocampus (4 images), PVWM (2 images) and SCWM (2 images) in the standardized regions described above on 5 consecutive coronal sections per animal were taken at 200x magnification with the same microscope and digital camera as above. Ki67-positive cells were counted using Image J software (Rasband, W.S., Image J US National Institutes of Health, Bethesda, Maryland, USA). The density (cells per mm^2^) of cleaved caspase-3 positive cells in the PVWM and SCWM were similarly measured. For the analysis in the hippocampus, the area fraction (%) of cleaved caspase-3 immuno-reactivity was measured using Leica QWin Pro V 3.5.1 software, since the hippocampus is densely populated with overlapping cleaved caspase-3 positive cells that could not be distinguished as single cells [35].

For the analysis of MBP and synaptophysin, digital images from the SCWM (2 images) for MBP and from the hippocampus (2 images in CA1/2 region, 2 images in CA3, 1 image in CA4 and DG) for the synaptophysin staining on 5 consecutive coronal sections per animal were acquired similarly at 200x magnification. In the PVWM no MBP immuno-reactivity was detected because of the developmental timing of appearance of late oligodendrocyte progenitor cells expressing MBP in the PVWM of sheep and humans [34,36,37]. The area fraction (%) of MBP and synaptophysin immuno-reactivity was measured using Leica QWin Pro V 3.5.1 software. All analyses were performed by a blinded observer.

### Data analysis

Data are expressed as mean ± SEM. Groups which were exposed at the same gestational age were compared using one-way ANOVA with Tukey or Dunnett’s tests for post-hoc analysis or by a non-parametric Kruskal-Wallis test as appropriate. Statistical analysis was performed by GraphPad Prism v5.0. Significance was accepted at p<0.05.

## Results

### MRI measurements

T2-weighted MRI images were used to measure anatomical variables such as brain diameter, cerebellum and pons diameter, corpus callosum (CC) length and CC and thalamus volume (Figure 1). Exposure to intra-amniotic LPS and/or maternal antenatal glucocorticoids either 7 or 14 days before delivery did not significantly alter any of the measurements in the fetal brain compared to control animals (Table 2). 

**Table 2:** Brain volumes measured with MRI images.

	Control (n=4)	7d Beta (n=6)	7d LPS (n=7)	Beta-Pre + 7d LPS (n=6)	14d LPS (n=7)	14d LPS + Beta-Post (n=7)
Brain (mm) (mid-sagittal antero-posterior)	20.0±1.1	18.4±0.9	23.4±3.7	18.4±0.7	19.2±0.7	22.5±0.5
Cerebellum (mm) (mid-sagittal cranio-caudal)	8.7±05	8.2±0.4	10.2±1.9	9.0±0.6	8.5±0.2	10.1±0.2
Pons (mm) (mid-sagittal antero-posterior)	2.3±0.2	2.2±0.1	2.7±0.4	2.3±0.1	2.3±0.1	2.8±0.1
Corpus callosum (mm) (mid-sagittal antero-posterior)	8.1±0.3	7.9±0.2	11.2±2.3	8.0±0.6	7.9±0.4	9.2±0.4
Corpus callosum (mm^3^)(mid-sagittal)	2.8±0.4	2.7±0.2	4.2±1.3	2.7±0.2	3.1±0.2	3.1±0.1
Thalamus (mm^3^) (maximal parasagittal)	8.5±1.4	8.9±0.8	12.1±2.6	10.1±0.7	9.4±0.6	9.3±0.5

Data corrected for body weight and expressed as mean ± SEM. LPS: lipopolysaccharide; Beta-Pre: betamethasone pre-treatment; Beta-Post: betamethasone post-treatment.

### Fetal exposure to intra-amniotic LPS and/or antenatal glucocorticoids 7 days before delivery

Brain inflammation was investigated by measuring the area fraction (%) of IBA1 and GFAP as markers for microglial and astrocyte infiltration respectively, in the fetal hippocampus, subcortical white matter and periventricular white matter. Apoptosis was assessed by immunohistochemical staining for cleaved caspase-3, proliferating cells were identified by Ki67 staining, myelination and late oligodendrocyte progenitor cells shown by MBP [36] and pre-synaptic vesicle density was assessed by synaptophysin staining. 

**Figure 2:**
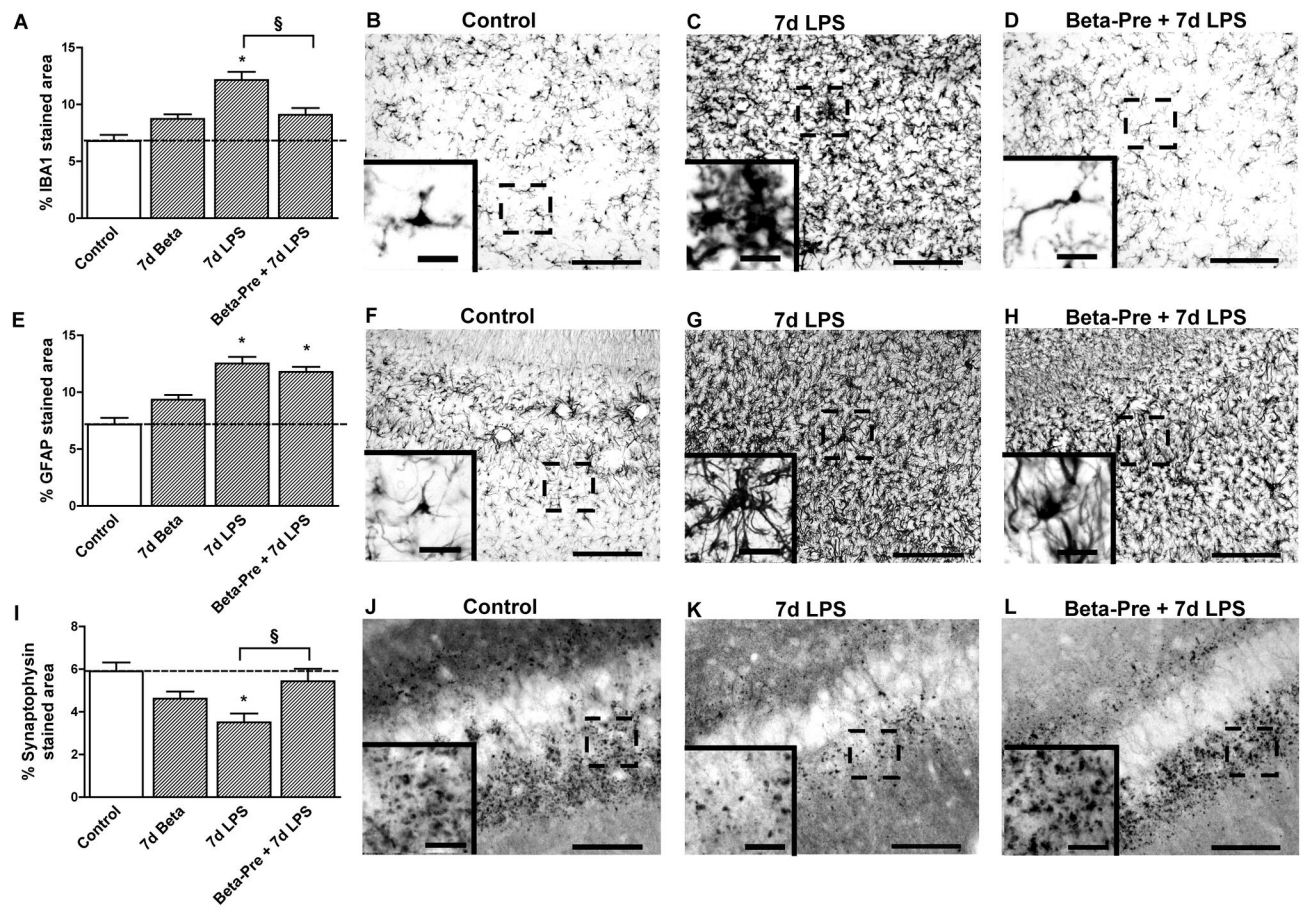
Hippocampal changes after intra-amniotic LPS exposure and antenatal glucocorticoids 7 days before delivery. **A**: The area fraction (%) of IBA1 immuno-reactivity increased significantly after intra-amniotic LPS exposure 7 days before delivery. **B**-**D**: Representative images of the IBA1 staining in the CA1 region of the hippocampus in controls (**B**), 7d LPS (**C**) and Beta-Pre + 7d LPS (**D**) exposed animals. **E**: Intra-amniotic LPS exposure significantly increased the hippocampal area fraction (%) of GFAP immuno-reactivity. **F**-**H**: Representative images of the GFAP staining in the CA1 region of the hippocampus in controls (**F**), 7d LPS (**G**) and Beta-Pre + 7d LPS (**H**) exposed animals. **I**: The area fraction (%) of synaptophysin immuno-reactivity decreased 7 days after intra-amniotic LPS exposure compared to controls. **J**-**L**: Representative images of the synaptophysin staining in the CA1 region of the hippocampus in controls (**J**), 7d LPS (**K**) and Beta-Pre + 7d LPS (L) exposed animals. Scale bar = 200 µm; scale bar insert = 25 µm. *p<0.05 versus controls and § p<0.05 between experimental groups using a one-way ANOVA with Tukey’s post hoc test.

**Figure 3:**
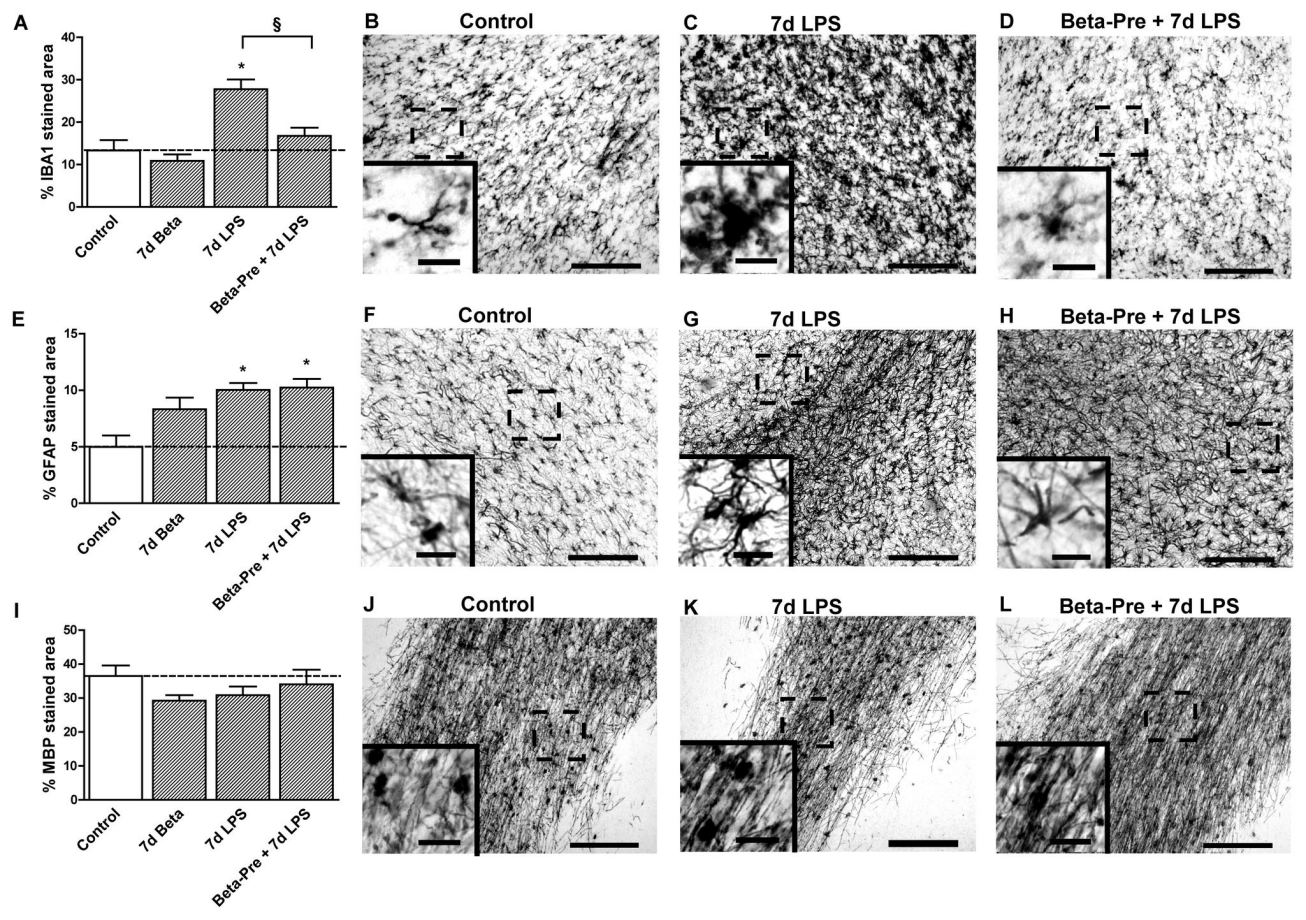
Effect of intra-amniotic LPS and antenatal glucocorticoid exposure 7 days before delivery on the subcortical white matter. **A**: The area fraction (%) of IBA1 immuno-reactivity in the subcortical white matter increased significantly after intra-amniotic LPS exposure 7 days before delivery. **B**-**D**: Representative images of the IBA1 staining in the SCWM in controls (**B**), 7d LPS (**C**) and Beta-Pre + 7d LPS (**D**) exposed animals. **E**: LPS exposure 7 days before delivery significantly increased the area fraction (%) of GFAP immuno-reactivity in the SCWM. **F**-**H**: Representative images of the GFAP staining in the SCWM in controls (**F**), 7d LPS (**G**) and Beta-Pre + 7d LPS (**H**) exposed animals. **I**: The area fraction (%) of MBP immuno-reactivity did not differ in animals exposed to intra-amniotic LPS and/or betamethasone exposure 7 days before delivery. **J**-**L**: Representative images of the MBP staining in the SCWM in controls (**J**), 7d LPS (**K**) and Beta-Pre + 7d LPS (L) exposed animals. Scale bar = 200 µm; scale bar insert = 25 µm. *p<0.05 versus controls and § p<0.05 between experimental groups using a one-way ANOVA with Tukey’s post hoc test.

**Figure 4:**
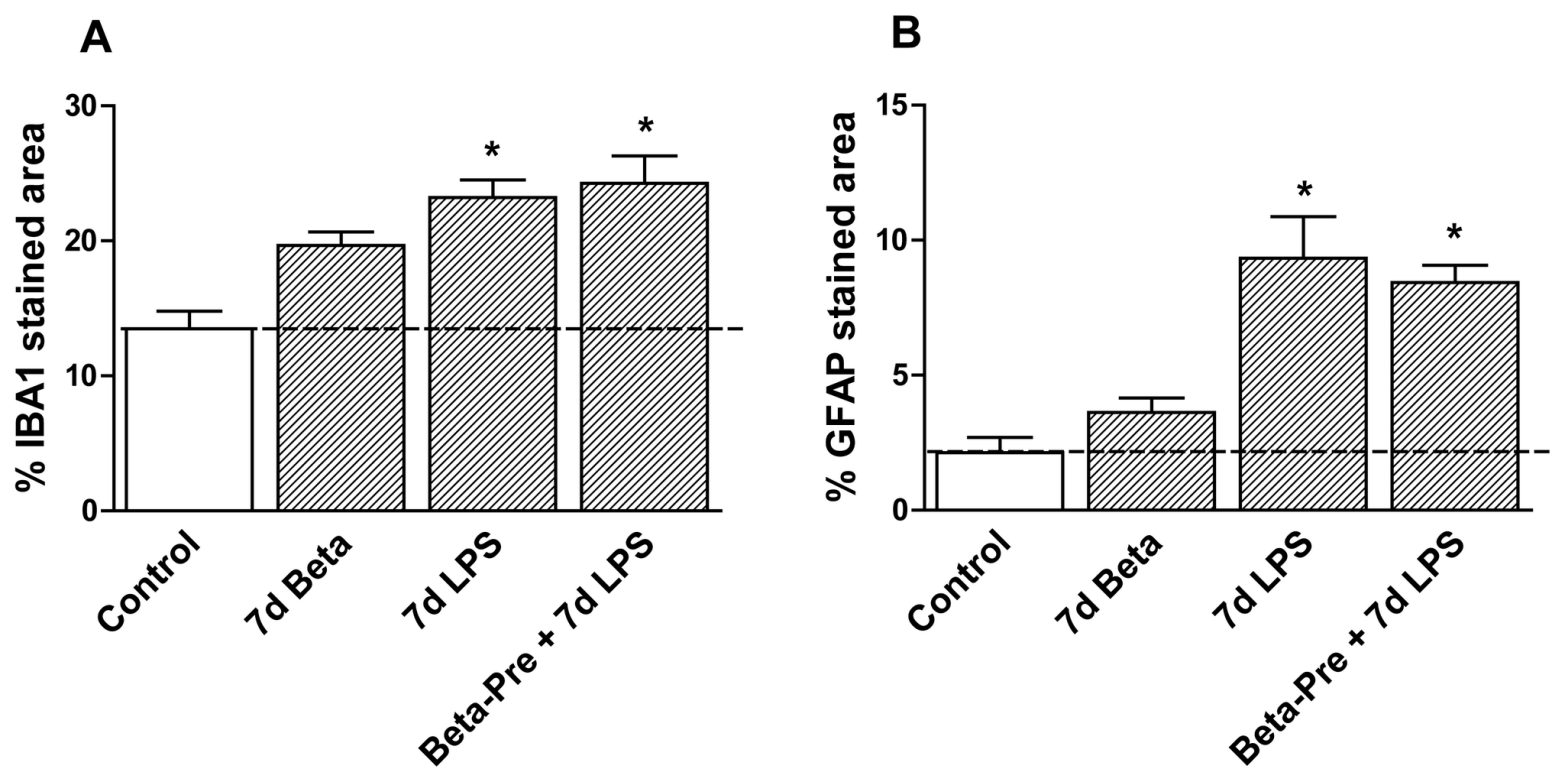
Periventricular white matter changes after intra-amniotic LPS and antenatal glucocorticoids 7 days before delivery. **A**: The area fraction (%) of IBA1 immuno-reactivity in the PVWM increased significantly in animals exposed to intra-amniotic LPS 7 days before delivery irrespective of the betamethasone pre-treatment. **B**: LPS exposure 7 days before delivery significantly increased the area fraction (%) of GFAP immuno-reactivity Irrespective of betamethasone pre-treatment. *p<0.05 versus controls using a one-way ANOVA with Tukey’s post hoc test.

Exposure to intra-amniotic LPS 7 days before delivery increased IBA1 immuno-reactivity in the hippocampus (Figure 2A), SCWM (Figure 3A) and PVWM (Figure 4A). Microglia of LPS exposed animals (Figure 2C, 3C) showed several morphological changes such as hypertrophic cell bodies and thickened shortened processes compared to controls (Figure 2B, 3B). GFAP immuno-reactivity increased significantly in all investigated brain regions in 7d LPS exposed animals (Figure 2E, 3E, 4B). Compared to controls (Figure 2F, 3F), GFAP-positive cells in the LPS exposed animals (Figure 2G, 3G) showed enlarged cell bodies and retracted processes suggesting active gliosis. Exposure to LPS 7 days before delivery increased apoptosis in the fetal hippocampus and PVWM compared to controls (Table 3) and significantly decreased the hippocampal area fraction (%) of synaptophysin immuno-reactivity (Figure 2I). LPS exposed animals (Figure 2K) showed a clear reduction in synaptophysin immuno-reactivity in the fetal hippocampus compared to controls (Figure 2J). Ki67 (Table 3) and MBP expression (Figure 3I) did not differ between 7d LPS exposed animals and control animals in the investigated brain regions. 

**Table 3:** Antenatal glucocorticoid treatment modulates the LPS-induced effects on apoptosis and proliferation in the fetal brain.

	Control	7d Beta	7d LPS	Beta-Pre + 7d LPS	14d LPS	14d LPS + Beta-Post
**Ki67 (cells/mm^2^)**						
Hippocampus	126±8	136±11	91±8	135±21	58±6*	145±7^£^
PVWM	71±9	71±11	54±5	55±15	66±10	70±6
SCWM	80±7	95±11	49±6	71±10	58±7	87±5**^£^**
**Cleaved caspase-3**						
Hippocampus (%stained area)	6.3±0.7	7.9±0.6	13.2±1.1*	10.9±1.0*	12.6±0.9*	9.8±0.7* ^£^
PVWM (cells/mm^2^)	325±30	296±31	463±34*	317±35**^*§*^**	269±20	482±43* ^£^
SCWM (cells/mm^2^)	94±13	152±12	137±17	121±13	138±13	201±21* ^£^

Data expressed as mean ± SEM. LPS: lipopolysaccharide; Beta-Pre: betamethasone pre-treatment; Beta-Post: betamethasone post-treatment. * p<0.05 versus controls; § p<0.05 versus 7d LPS; £ p<0.05 versus 14d LPS

Betamethasone pre-treatment at 106 days gestational age before intra-amniotic LPS exposure at 113 days gestational age (Beta-Pre + 7d LPS) reduced IBA1 immuno-reactivity in the fetal hippocampus (Figure 2D) and SCWM (Figure 3D) compared to 7d LPS exposed animals. Betamethasone pre-treatment before the LPS exposure normalized synaptophysin immuno-reactivity (Figure 2I) in the hippocampus but did not prevent the LPS-induced increase in GFAP immuno-reactivity in the investigated regions of the fetal brain (Figure 2H, 3H, 4B) nor did it prevent increased hippocampal apoptosis (Table 3). 

### Fetal exposure to intra-amniotic LPS and/or antenatal glucocorticoids 14 days before delivery

**Figure 5:**
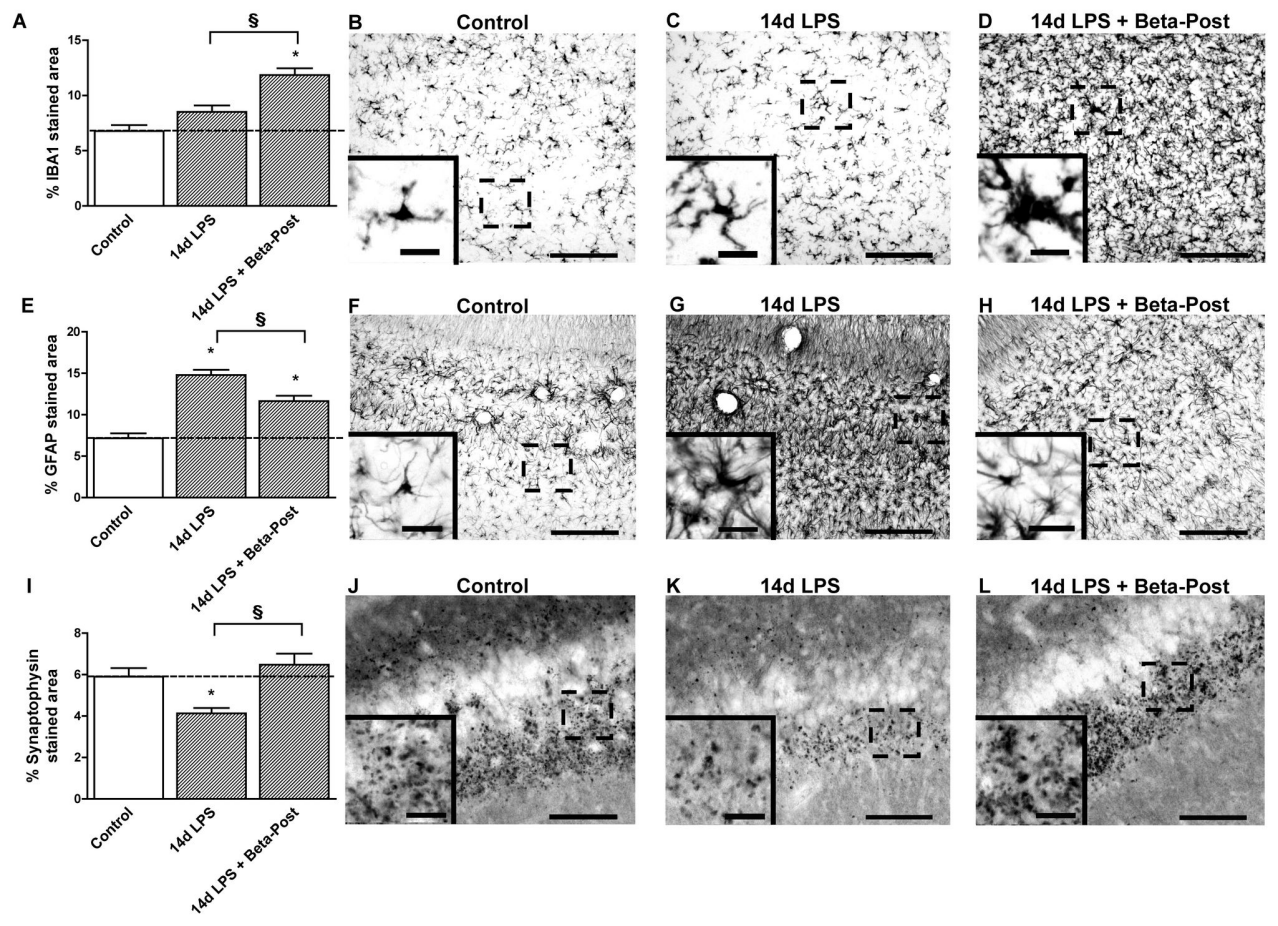
Hippocampal changes after intra-amniotic LPS exposure and antenatal glucocorticoids 14 days before delivery. **A**: The area fraction (%) of IBA1 immuno-reactivity increased after exposure to intra-amniotic LPS exposure followed by Betamethasone post-treatment. **B**-**D**: Representative images of the IBA1 staining in the CA1 region of the hippocampus in controls (**B**), 14d LPS (**C**) and 14d LPS + Beta-Post (**D**) exposed animals. **E**: Intra-amniotic LPS exposure increased GFAP immuno-reactivity in the hippocampus. **F**-**H**: Representative images of the GFAP staining in the CA1 region of the hippocampus in controls (**F**), 14d LPS (**G**), and 14d LPS + Beta-Post (**H**) exposed animals. **I**: The area fraction (%) of synaptophysin immuno-reactivity decreased 14 days after intra-amniotic LPS exposure compared to controls. **J**-**L**: Representative images of the synaptophysin staining in the CA1 region of the hippocampus in controls (**J**), 14d LPS (**K**) and 14d LPS + Beta-Post (**L**) exposed animals. Scale bar = 200 µm; scale bar insert = 25 µm. *p<0.05 versus controls and § p<0.05 between experimental groups using a one-way ANOVA with Tukey’s post hoc test.

**Figure 6:**
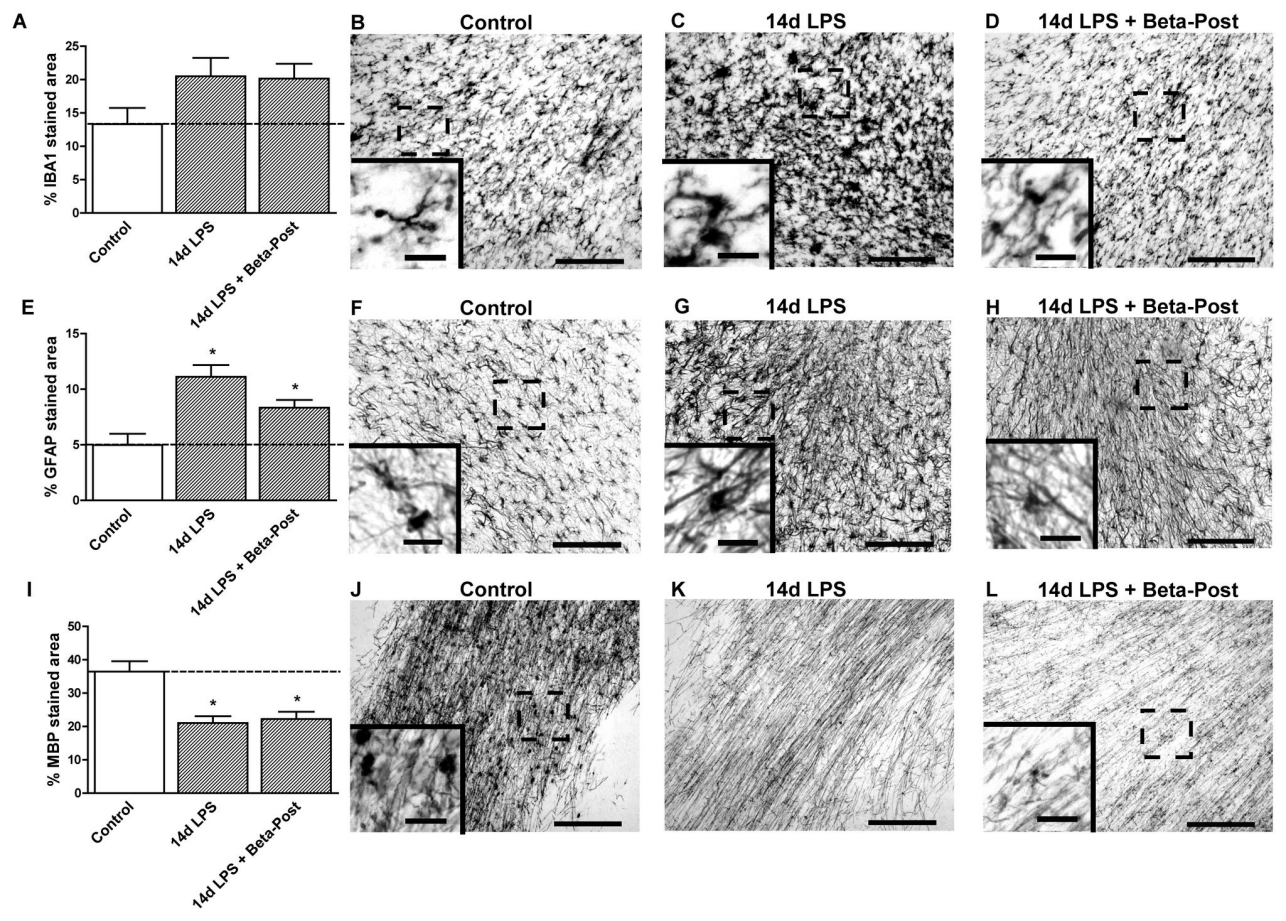
Effect of intra-amniotic LPS and antenatal glucocorticoid exposure 14 days before delivery on the subcortical white matter. **A**: The area fraction (%) of IBA1 immuno-reactivity did not change after intra-amniotic LPS and/or betamethasone exposure 14 days before delivery. **B**-**D**: Representative images of the IBA1 staining in the SCWM in controls (**B**), 14d LPS (**C**) and 14d LPS + Beta-Post (**D**) exposed animals. **E**: LPS exposure 14 days before delivery significantly increased the area fraction (%) of GFAP immuno-reactivity irrespective of betamethasone post-treatment. **F**-**H**: Representative images of the GFAP staining in the SCWM in controls (**F**), 14d LPS (**G**) and 14d LPS + Beta-Post (**H**) exposed animals. **I**: The area fraction (%) of MBP immuno-reactivity decreased 14 days after intra-amniotic LPS exposure irrespective of betamethasone treatment compared to controls. **J**-**L**: Representative images of the MBP staining in the SCWM in controls (**J**), 14d LPS (**K**) and 14d LPS + Beta-Post (**L**) exposed animals. Scale bar = 200 µm; scale bar insert = 25 µm. *p<0.05 versus controls using a one-way ANOVA with Tukey’s post hoc test.

**Figure 7:**
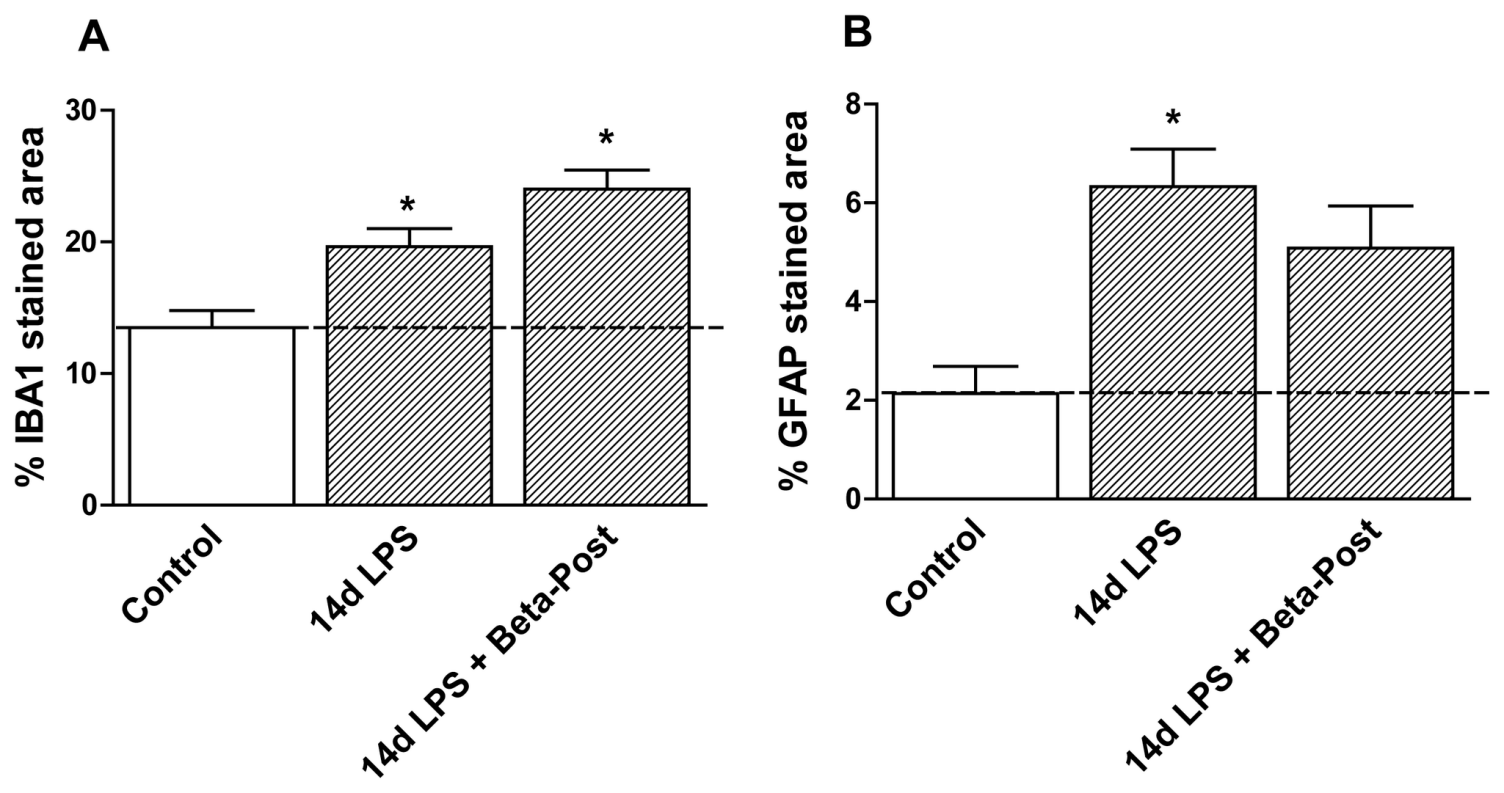
Periventricular white matter changes after intra-amniotic LPS and antenatal glucocorticoids 14 days before delivery. **A**: The area fraction (%) of IBA1 immuno-reactivity in the PVWM increased after intra-amniotic LPS exposure 14 days before delivery irrespective of the betamethasone post-treatment. **B**: LPS exposure 14 days before delivery increased the area fraction (%) of GFAP immuno-reactivity. *p<0.05 versus controls using a one-way ANOVA with Tukey’s post hoc test.

Exposure to intra-amniotic LPS 14 days before delivery (14d LPS) increased IBA1 immuno-reactivity in the PVWM and GFAP immuno-reactivity in all investigated regions (Figure 5E, 6E, 7) compared to control animals, with morphological changes consistent with activation of microglia and astrocytes (Figure 5C). Animals exposed to LPS 14 days before delivery showed reduced hippocampal synaptophysin expression (Figure 5I) and less MBP immuno-reactivity and loss of MBP-positive cells in the SCWM (Figure 6I) compared to control animals indicating grey and white matter injury. Proliferation rates were decreased and apoptosis was increased in the hippocampus in 14d LPS exposed animals compared to controls (Table 3).

Animals that received betamethasone post-treatment 7 days after the LPS exposure (14d LPS + Beta-Post) showed increased IBA1 immuno-reactivity in the fetal hippocampus compared to animals exposed to LPS only (Figure 5D). Betamethasone post-treatment decreased hippocampal GFAP immuno-reactivity which was induced by the LPS exposure (Figure 5H) and normalized hippocampal synaptophysin expression (Figure 5L). LPS-induced white matter injury was not prevented by Betamethasone post-treatment and was still evident in the 14d LPS + Beta-Post animals (Figure 6L) with decreased MBP immuno-reactivity in the SCWM. When exposed to both LPS and Betamethasone post-treatment, animals showed increased numbers of Ki67-positive cells in the hippocampus and SCWM and increased apoptosis in the PVWM and SCWM compared to animals exposed to LPS only (Table 3). Betamethasone post-treatment did decrease the LPS-induced apoptosis in the fetal hippocampus (Table 3). 

## Discussion

We investigated the effects of intra-uterine inflammation and antenatal glucocorticoids, two common antenatal exposures for preterm infants at two different time point in gestation, on the ovine fetal brain. Intra-amniotic LPS exposure either at 106d (14d LPS exposure) or 113d GA (7d LPS exposure) before preterm delivery at 120d GA induced a cerebral inflammatory response with hippocampal and white matter injury. Glucocorticoid treatment in the absence of intra-uterine inflammation did not affect the fetal brain. Antenatal maternal glucocorticoids administered with pre-existing intra-amniotic inflammation aggravated the LPS-induced inflammatory changes in the fetal brain. Antenatal glucocorticoids given prior to LPS reduced the effects of intra-uterine inflammation on the fetal brain. Although this study only describes the interactive effects of LPS and glucocorticoids at two different time-points during gestation, these findings do identify differential effects of these two common exposures on the fetal near term brain. 

Our findings did not show any harmful effects of antenatal glucocorticoid treatment in the absence of intra-uterine inflammation on the fetal brain. Although antenatal glucocorticoids reduce the risk of intraventricular hemorrhage and periventricular leukomalacia [9,38], there are concerns about the effects of glucocorticoids on fetal brain development, in particular on brain growth [39,40], neurogenesis [41,42] and myelination [43]. Recently, Tijsseling et al. [44] showed reduced histological neuronal density in the hippocampus of neonates after antenatal glucocorticoid treatment. In animal models multiple doses of antenatal glucocorticoids at different time points during gestation can modulate apoptotic rates [45]. Furthermore, repeated doses of antenatal glucocorticoids decrease the expression of cytoskeletal proteins microtubule-associated protein (MAP)1B and MAP2 and synaptophysin expression in fetal sheep brain suggesting functional disturbances in the neuronal network [46,47]. Although it is less clear how a single course of antenatal glucocorticoids affects the developing brain, in clinical practice the beneficial effects of glucocorticoids on lung maturation and consequent neonatal survival outweigh these concerns.

We used T2-weighted MRI imaging to investigate volumetric changes in the ovine fetal brain. The MRI imaging did not detect any gross volumetric changes after exposure to intra-amniotic LPS which is in line with previous clinical findings [48,49]. Acute (7d before delivery) or persistent (14d before delivery) exposure to intra-uterine inflammation induced an inflammatory response in the brain regions we investigated as indicated by increased microglia and astrogliosis associated with hypomyelination and reduced synaptophysin expression. We did not monitor brain function, but microglial activation and apoptosis in the white matter of preterm lambs exposed to LPS correlated with increased EEG delta frequency which was associated with seizure disorders [24,50]. Studies of Dean et al.[51] and Keogh et al.[52] support these findings by showing that inflammatory changes in the ovine fetal brain correlated with loss of the normal maturational increase in the EEG amplitude. Furthermore, our findings of decreased pre-synaptic vesicle density in the fetal hippocampus suggest that synaptic transmission was compromised after LPS exposure [53].

We investigated the effect of the timing of combined exposures of the ovine near term brain to intra-amniotic inflammation and antenatal glucocorticoids, since glucocorticoids can have both pro- and anti-inflammatory properties in the presence of inflammation [54]. Antenatal glucocorticoids are administered when preterm birth is imminent irrespective of the cause of preterm birth [9]. However, there is a lack of knowledge concerning the use of antenatal glucocorticoids in the settings of intra-uterine infections [9]. Kallapur et al. [55] previously showed that combined maternal betamethasone and intra-amniotic LPS exposure increased fetal lung inflammation compared to LPS exposure alone suggesting that glucocorticoids alter the immune response of the preterm lung to inflammation. In this model antenatal exposure to glucocorticoids either before or after an inflammatory challenge has contrasting effects on the fetal immune response [25,26] and subsequent brain inflammation and injury. Antenatal glucocorticoid administration before the LPS exposure prevented inflammation in the fetal lung, thymus and brain whereas glucocorticoid administration after the LPS exposure aggravated inflammation in the fetal organs [25,26]. Although the anti-inflammatory actions of glucocorticoids have been recognized for several decades, recent studies suggest that glucocorticoids can also have pro-inflammatory properties [54,56]. The balance of these pro- and anti-inflammatory effects depends on which phase of the immune response the glucocorticoids are administered. Glucocorticoid-mediated activation of the glucocorticoid receptor induces suppression of transcriptional activity of NF-ĸB and other genes involved in the inflammatory response [57,58]. Therefore pre-treatment with glucocorticoids inhibits the subsequent inflammatory response initiated by LPS-mediated Toll-like receptor signaling, resulting in anti-inflammatory immunological response. However, when the inflammatory response is initiated before activation of the glucocorticoid receptor, the immune response may shift to pro-inflammatory as glucocorticoids can enhance the expression of Toll-like receptors and consequently increase pro-inflammatory cytokine production [59,60]. Consequently, the timing of exposure to anti- and pro-inflammatory stimuli appears to be essential in determining the eventual outcome of the immune response, not only *in vitro*, but also *in vivo* in the fetus. 

There are limitations to this study. We were not able to identify which cells in the fetal brain underwent apoptosis or loss of proliferation after the exposures. Furthermore, we did not assess what the effects of these histological changes were on the functional outcome of the fetal brain. However, recent studies have shown that minor changes in the activation state of microglia or moderate white matter damage can affect EEG maturation in the fetal brain suggesting that even low grade inflammation/injury as seen in this study can impact fetal brain function [24,51,52]. The animals in this study were exposed to intra-amniotic LPS and maternal glucocorticoids either 7 days or 14 days before preterm delivery. However, exposures at different time points, for different intervals and to a single dose or repeated doses of glucocorticoids during fetal development may have different outcomes. Although our study does not identify new concerns for the use of a single dose of glucocorticoids, long term effects of antenatal glucocorticoid treatment on neonatal neurodevelopment remain unclear. 

In conclusion, we have shown that intra-amniotic LPS exposure either at 106d GA or 113d GA induced inflammation and injury in the regions of the ovine near term brain that we evaluated and without volumetric changes measured by MRI. Our findings demonstrate that maternal antenatal betamethasone administration can increase the inflammatory changes in the fetal brain caused by pre-existing intra-amniotic inflammation suggesting that when a fetus is exposed to chorioamnionitis, the brain may sustain additional injury after a secondary exposure to maternal glucocorticoids. Antenatal glucocorticoids given prior to intra-amniotic inflammation reduced the cerebral inflammatory response after intra-amniotic LPS and prevented hippocampal and white matter injury in the fetal brain. This study shows that the timing of treatment with maternal glucocorticoids in the setting of intra-uterine inflammation determines the effects on the near term fetal brain. Therefore, future experimental studies should focus on the immuno-modulatory effects of antenatal glucocorticoid treatment at the onset of chorioamnionitis. 

## References

[1] Goldenberg RL, Hauth JC, Andrews WW (2000) Intrauterine infection and preterm delivery. N Engl J Med 342: 1500-1507. doi:10.1056/NEJM200005183422007. PubMed: 10816189. 10816189

[2] Redline RW (2004) Placental inflammation. Semin Neonatol 9: 265-274. doi:10.1016/j.siny.2003.09.005. PubMed: 15251143. 15251143

[3] Hartling L, Liang Y, Lacaze-Masmonteil T (2012) Chorioamnionitis as a risk factor for bronchopulmonary dysplasia: a systematic review and meta-analysis. Arch Dis Child Fetal Neonatal Ed 97: F8-F17. doi:10.1136/adc.2010.210187. PubMed: 21697236. 21697236

[4] Kallapur SG, Willet KE, Jobe AH, Ikegami M, Bachurski CJ (2001) Intra-amniotic endotoxin: chorioamnionitis precedes lung maturation in preterm lambs. Am J Physiol Lung Cell Mol Physiol 280: L527-L536. PubMed: 11159037. 11159037 10.1152/ajplung.2001.280.3.L527

[5] Wolfs TG, Buurman WA, Zoer B, Moonen RM, Derikx JP et al. (2009) Endotoxin induced chorioamnionitis prevents intestinal development during gestation in fetal sheep. PLOS ONE 4: e5837. doi:10.1371/journal.pone.0005837. PubMed: 19503810. 19503810 PMC2688751

[6] Been JV, Lievense S, Zimmermann LJ, Kramer BW, Wolfs TG (2013) Chorioamnionitis as a risk factor for necrotizing enterocolitis: a systematic review and meta-analysis. J Pediatr 162: 236-242 e232 22920508 10.1016/j.jpeds.2012.07.012

[7] Shatrov JG, Birch SC, Lam LT, Quinlivan JA, McIntyre S et al. (2010) Chorioamnionitis and cerebral palsy: a meta-analysis. Obstet Gynecol 116: 387-392. doi:10.1097/AOG.0b013e3181e90046. PubMed: 20664400. 20664400

[8] Gantert M, Been JV, Gavilanes AWD, Garnier Y, Zimmermann LJI et al. (2010) Chorioamnionitis: a multiorgan disease of the fetus? J Perinatol 30 Suppl: 21-30. doi:10.1038/jp.2010.96. PubMed: 20877404. 20877404

[9] Been JV, Degraeuwe PL, Kramer BW, Zimmermann LJ (2011) Antenatal steroids and neonatal outcome after chorioamnionitis: a meta-analysis. BJOG 118: 113-122. doi:10.1111/j.1471-0528.2010.02751.x. PubMed: 21054759. 21054759

[10] Roberts D, Dalziel S (2006) Antenatal corticosteroids for accelerating fetal lung maturation for women at risk of preterm birth. Cochrane Database Syst Rev: CD: 004454 PubMed: 16856047. 10.1002/14651858.CD004454.pub2 16856047

[11] Malaeb S, Dammann O (2009) Fetal inflammatory response and brain injury in the preterm newborn. J Child Neurol 24: 1119-1126. doi:10.1177/0883073809338066. PubMed: 19605775. 19605775 PMC3695470

[12] Dammann O, Leviton A (1997) Maternal intrauterine infection, cytokines, and brain damage in the preterm newborn. Pediatr Res 42: 1-8. doi:10.1203/00006450-199707000-00001. PubMed: 9212029. 9212029

[13] Bashiri A, Burstein E, Mazor M (2006) Cerebral palsy and fetal inflammatory response syndrome: a review. J Perinat Med 34: 5-12. PubMed: 16489880. 16489880 10.1515/JPM.2006.001

[14] Grether JK, Nelson KB, Walsh E, Willoughby RE, Redline RW (2003) Intrauterine exposure to infection and risk of cerebral palsy in very preterm infants. Arch Pediatr Adolesc Med 157: 26-32. doi:10.1001/archpedi.157.1.26. PubMed: 12517191. 12517191

[15] Hendson L, Russell L, Robertson CMT, Liang Y, Chen Y et al. (2011) Neonatal and neurodevelopmental outcomes of very low birth weight infants with histologic chorioamnionitis. J Pediatr 158: 397-402. doi:10.1016/j.jpeds.2010.09.010. PubMed: 20961565. 20961565

[16] Limperopoulos C, Bassan H, Sullivan NR, Soul JS, Robertson RL et al. (2008) Positive screening for autism in ex-preterm infants: prevalence and risk factors. Pediatrics 121: 758-765. doi:10.1542/peds.2007-2158. PubMed: 18381541. 18381541 PMC2703587

[17] Hagberg H, Gressens P, Mallard C (2012) Inflammation during fetal and neonatal life: implications for neurologic and neuropsychiatric disease in children and adults. Ann Neurol 71: 444-457. doi:10.1002/ana.22620. PubMed: 22334391. 22334391

[18] Buka SL, Tsuang MT, Torrey EF, Klebanoff MA, Bernstein D et al. (2001) Maternal infections and subsequent psychosis among offspring. Arch Gen Psychiatry 58: 1032-1037. doi:10.1001/archpsyc.58.11.1032. PubMed: 11695949. 11695949

[19] Gantert M, Kreczmanski P, Kuypers E, Jellema R, Strackx E et al. (2012) Effects of in utero endotoxemia on the ovine fetal brain: a model for schizophrenia? Front Biosci (Elite Ed) 4: 2845-2853. PubMed: 22652683. 10.2741/e588 22652683

[20] Yoon BH, Kim CJ, Romero R, Jun JK, Park KH et al. (1997) Experimentally induced intrauterine infection causes fetal brain white matter lesions in rabbits. Am J Obstet Gynecol 177: 797-802. doi:10.1016/S0002-9378(97)70271-0. PubMed: 9369822. 9369822

[21] Burd I, Brown A, Gonzalez JM, Chai J, Elovitz MA (2011) A mouse model of term chorioamnionitis: unraveling causes of adverse neurological outcomes. Reprod Sci 18: 900-907. doi:10.1177/1933719111398498. PubMed: 21421895. 21421895 PMC3343123

[22] Back SA, Riddle A, Dean J, Hohimer AR (2012) The instrumented fetal sheep as a model of cerebral white matter injury in the premature infant. Neurotherapeutics 9: 359-370. doi:10.1007/s13311-012-0108-y. PubMed: 22399133. 22399133 PMC3337024

[23] Nitsos I, Rees SM, Duncan J, Kramer BW, Harding R et al. (2006) Chronic exposure to intra-amniotic lipopolysaccharide affects the ovine fetal brain. J Soc Gynecol Investig 13: 239-247. doi:10.1016/j.jsgi.2006.02.011. PubMed: 16697939. 16697939

[24] Gavilanes AW, Gantert M, Strackx E, Zimmermann LJ, Seeldrayers S et al. (2010) Increased EEG delta frequency corresponds to chorioamnionitis-related brain injury. Front Biosci (Schol Ed) 2: 432-438. PubMed: 20036959. 20036959 10.2741/s76

[25] Kuypers E, Collins JJ, Jellema RK, Wolfs TG, Kemp MW et al. (2012) Ovine fetal thymus response to lipopolysaccharide-induced chorioamnionitis and antenatal corticosteroids. PLOS ONE 7: e38257. doi:10.1371/journal.pone.0038257. PubMed: 22693607. 22693607 PMC3365024

[26] Kuypers E, Collins JJP, Kramer BW, Ofman G, Nitsos I et al. (2012) Intra-amniotic LPS and antenatal betamethasone: inflammation and maturation in preterm lamb lungs. Am J Physiol Lung Cell Mol Physiol 302: 380-389. doi:10.1152/ajplung.00338.2011. PubMed: 22160306. PMC3289264 22160306

[27] Kramer BW, Joshi SN, Moss TJ, Newnham JP, Sindelar R et al. (2007) Endotoxin-induced maturation of monocytes in preterm fetal sheep lung. Am J Physiol Lung Cell Mol Physiol 293: L345-L353. doi:10.1152/ajplung.00003.2007. PubMed: 17513458. 17513458

[28] Collins JJ, Kuypers E, Nitsos I, Jane Pillow J, Polglase GR et al. (2012) LPS-induced chorioamnionitis and antenatal corticosteroids modulate Shh signaling in the ovine fetal lung. Am J Physiol Lung Cell Mol Physiol 303: L778-L787. doi:10.1152/ajplung.00280.2011. PubMed: 22962010. 22962010 PMC3517680

[29] Ballard PL, Ballard RA (1995) Scientific basis and therapeutic regimens for use of antenatal glucocorticoids. Am J Obstet Gynecol 173: 254-262. doi:10.1016/0002-9378(95)90210-4. PubMed: 7631700. 7631700

[30] Rebello CM, Ikegami M, Polk DH, Jobe AH (1996) Postnatal lung responses and surfactant function after fetal or maternal corticosteroid treatment. J Appl Physiol (1985) 80: 1674-1680. doi:10.1063/1.362966. PubMed: 8727554. 8727554

[31] Jobe AH, Newnham J, Willet K, Sly P, Ikegami M (1998) Fetal versus maternal and gestational age effects of repetitive antenatal glucocorticoids. Pediatrics 102: 1116-1125. doi:10.1542/peds.102.5.1116. PubMed: 9794943. 9794943

[32] McIntosh GH, Baghurst KI, Potter BJ, Hetzel BS (1979) Foetal brain development in the sheep. Neuropathol Appl Neurobiol 5: 103-114. doi:10.1111/j.1365-2990.1979.tb00664.x. PubMed: 471183. 471183

[33] Roelfsema V, Bennet L, George S, Wu D, Guan J et al. (2004) Window of opportunity of cerebral hypothermia for postischemic white matter injury in the near-term fetal sheep. J Cereb Blood Flow Metab 24: 877-886. PubMed: 15362718. 15362718 10.1097/01.WCB.0000123904.17746.92

[34] Jellema RK, Passos VL, Zwanenburg A, Ophelders DR, De Munter S et al. (2013) Cerebral inflammation and mobilization of the peripheral immune system following global hypoxic-ischemia in preterm sheep. J Neuroinflammation 10: 13. doi:10.1186/1742-2094-10-13. PubMed: 23347579. 23347579 PMC3614445

[35] Yawno T, Hirst JJ, Castillo-Melendez M, Walker DW (2009) Role of neurosteroids in regulating cell death and proliferation in the late gestation fetal brain. Neuroscience 163: 838-847. doi:10.1016/j.neuroscience.2009.07.009. PubMed: 19591903. 19591903

[36] Back SA, Luo NL, Borenstein NS, Levine JM, Volpe JJ et al. (2001) Late oligodendrocyte progenitors coincide with the developmental window of vulnerability for human perinatal white matter injury. J Neurosci 21: 1302-1312. PubMed: 11160401. 11160401 10.1523/JNEUROSCI.21-04-01302.2001 PMC6762224

[37] Back SA, Riddle A, Hohimer AR (2006) Role of instrumented fetal sheep preparations in defining the pathogenesis of human periventricular white-matter injury. J Child Neurol 21: 582-589. doi:10.1177/08830738060210070101. PubMed: 16970848. 16970848

[38] Baud O, Foix-L'Helias L, Kaminski M, Audibert F, Jarreau PH et al. (1999) Antenatal glucocorticoid treatment and cystic periventricular leukomalacia in very premature infants. N Engl J Med 341: 1190-1196. doi:10.1056/NEJM199910143411604. PubMed: 10519896. 10519896

[39] Modi N, Lewis H, Al-Naqeeb N, Ajayi-Obe M, Doré CJ et al. (2001) The effects of repeated antenatal glucocorticoid therapy on the developing brain. Pediatr Res 50: 581-585. doi:10.1203/00006450-200111000-00008. PubMed: 11641451. 11641451

[40] French NP, Hagan R, Evans SF, Godfrey M, Newnham JP (1999) Repeated antenatal corticosteroids: size at birth and subsequent development. Am J Obstet Gynecol 180: 114-121. doi:10.1016/S0002-9378(99)70160-2. PubMed: 9914589. 9914589

[41] Uno H, Lohmiller L, Thieme C, Kemnitz JW, Engle MJ et al. (1990) Brain damage induced by prenatal exposure to dexamethasone in fetal rhesus macaques. I. Hippocampus - Brain Res Dev Brain Res 53: 157-167. doi:10.1016/0165-3806(90)90002-G. 2357788

[42] Noorlander CW, Visser GH, Ramakers GM, Nikkels PG, de Graan PN (2008) Prenatal corticosteroid exposure affects hippocampal plasticity and reduces lifespan. Dev Neurobiol 68: 237-246. doi:10.1002/dneu.20583. PubMed: 18000831. 18000831

[43] Dunlop SA, Archer MA, Quinlivan JA, Beazley LD, Newnham JP (1997) Repeated prenatal corticosteroids delay myelination in the ovine central nervous system. J Matern Fetal Med 6: 309-313. doi:10.3109/14767059709162011. PubMed: 9438210. 9438210

[44] Tijsseling D, Wijnberger LD, Derks JB, van Velthoven CT, de Vries WB et al. (2012) Effects of antenatal glucocorticoid therapy on hippocampal histology of preterm infants. PLOS ONE 7: e33369. doi:10.1371/journal.pone.0033369. PubMed: 22457757. 22457757 PMC3311632

[45] Malaeb SN, Hovanesian V, Sarasin MD, Hartmann SM, Sadowska GB et al. (2009) Effects of maternal antenatal glucocorticoid treatment on apoptosis in the ovine fetal cerebral cortex. J Neurosci Res 87: 179-189. doi:10.1002/jnr.21825. PubMed: 18711727. 18711727 PMC2692887

[46] Antonow-Schlorke I, Müller T, Brodhun M, Wicher C, Schubert H et al. (2007) Betamethasone-related acute alterations of microtubule-associated proteins in the fetal sheep brain are reversible and independent of age during the last one-third of gestation. Am J Obstet Gynecol 196: 553: e551-e556. PubMed: 17547892. 10.1016/j.ajog.2006.10.898 17547892

[47] Antonow-Schlorke I, Kühn B, Müller T, Schubert H, Sliwka U et al. (2001) Antenatal betamethasone treatment reduces synaptophysin immunoreactivity in presynaptic terminals in the fetal sheep brain. Neurosci Lett 297: 147-150. doi:10.1016/S0304-3940(00)01605-0. PubMed: 11137749. 11137749

[48] Kaukola T, Herva R, Perhomaa M, Pääkkö E, Kingsmore S et al. (2006) Population cohort associating chorioamnionitis, cord inflammatory cytokines and neurologic outcome in very preterm, extremely low birth weight infants. Pediatr Res 59: 478-483. Available online at: doi:10.1203/01.pdr.0000182596.66175.ee. PubMed: 16492993. 16492993 10.1203/01.pdr.0000182596.66175.ee

[49] Chau V, Poskitt KJ, McFadden DE, Bowen-Roberts T, Synnes A et al. (2009) Effect of chorioamnionitis on brain development and injury in premature newborns. Ann Neurol 66: 155-164. doi:10.1002/ana.21713. PubMed: 19743455. 19743455

[50] Gavilanes AWD, Strackx E, Kramer BW, Gantert M, Van den Hove D et al. (2009) Chorioamnionitis induced by intraamniotic lipopolysaccharide resulted in an interval-dependent increase in central nervous system injury in the fetal sheep. Am J Obstet Gynecol 200: 1-8. doi:10.1016/S0002-9378(09)00292-0. PubMed: 19121654. 19217590

[51] Dean JM, van de Looij Y, Sizonenko SV, Lodygensky GA, Lazeyras F et al. (2011) Delayed cortical impairment following lipopolysaccharide exposure in preterm fetal sheep. Ann Neurol 70: 846-856. doi:10.1002/ana.22480. PubMed: 22002627. 22002627

[52] Keogh MJ, Bennet L, Drury PP, Booth LC, Mathai S et al. (2012) Subclinical exposure to low-dose endotoxin impairs EEG maturation in preterm fetal sheep. Am J Physiol Regul Integr Comp Physiol 303: 270-278. doi:10.1152/ajpregu.00216.2012. PubMed: 22696578. 22696578

[53] Valtorta F, Pennuto M, Bonanomi D, Benfenati F (2004) Synaptophysin: leading actor or walk-on role in synaptic vesicle exocytosis? BioEssays 26: 445-453. doi:10.1002/bies.20012. PubMed: 15057942. 15057942

[54] Busillo JM, Cidlowski JA (2013) The five Rs of glucocorticoid action during inflammation: ready, reinforce, repress, resolve, and restore. Trends Endocrinol Metab, 24: 109–19. PubMed: 23312823. 23312823 10.1016/j.tem.2012.11.005 PMC3667973

[55] Kallapur SG, Kramer BW, Moss TJ, Newnham JP, Jobe AH et al. (2003) Maternal glucocorticoids increase endotoxin-induced lung inflammation in preterm lambs. Am J Physiol Lung Cell Mol Physiol 284: L633-L642. PubMed: 12471018. 12471018 10.1152/ajplung.00344.2002

[56] Rao NA, McCalman MT, Moulos P, Francoijs KJ, Chatziioannou A et al. (2011) Coactivation of GR and NFKB alters the repertoire of their binding sites and target genes. Genome Res 21: 1404-1416. doi:10.1101/gr.118042.110. PubMed: 21750107. 21750107 PMC3166826

[57] Liden J, Delaunay F, Rafter I, Gustafsson J, Okret S (1997) A new function for the C-terminal zinc finger of the glucocorticoid receptor. Repression of RelA transactivation. J Biol Chem 272: 21467-21472. doi:10.1074/jbc.272.34.21467. PubMed: 9261164. 9261164

[58] Beck IM, Vanden Berghe W, Vermeulen L, Yamamoto KR, Haegeman G et al. (2009) Crosstalk in inflammation: the interplay of glucocorticoid receptor-based mechanisms and kinases and phosphatases. Endocr Rev 30: 830-882. doi:10.1210/er.2009-0013. PubMed: 19890091. 19890091 PMC2818158

[59] Chinenov Y, Rogatsky I (2007) Glucocorticoids and the innate immune system: crosstalk with the toll-like receptor signaling network. Mol Cell Endocrinol 275: 30-42. doi:10.1016/j.mce.2007.04.014. PubMed: 17576036. 17576036

[60] Busillo JM, Azzam KM, Cidlowski JA (2011) Glucocorticoids sensitize the innate immune system through regulation of the NLRP3 inflammasome. J Biol Chem 286: 38703-38713. doi:10.1074/jbc.M111.275370. PubMed: 21940629. 21940629 PMC3207479

